# Simulation of Water Environmental Capacity and Pollution Load Reduction Using QUAL2K for Water Environmental Management 

**DOI:** 10.3390/ijerph9124504

**Published:** 2012-12-07

**Authors:** Ruibin Zhang, Xin Qian, Xingcheng Yuan, Rui Ye, Bisheng Xia, Yulei Wang

**Affiliations:** State Key Laboratory of Pollution Control and Resource Reuse, School of the Environment, Nanjing University, Nanjing 210046, China; E-Mails: zhangrb88@126.com (R.Z.); yxingcheng@163.com (X.Y.); rui.ye0220@163.com (R.Y.); xiabisheng1984@163.com (B.X.); gywyl127@163.com (Y.W.)

**Keywords:** Hongqi River, pollution load reduction, QUAL2K model, simulation, water environmental capacity, water environmental management

## Abstract

In recent years, water quality degradation associated with rapid socio-economic development in the Taihu Lake Basin, China, has attracted increasing attention from both the public and the Chinese government. The primary sources of pollution in Taihu Lake are its inflow rivers and their tributaries. Effective water environmental management strategies need to be implemented in these rivers to improve the water quality of Taihu Lake, and to ensure sustainable development in the region. The aim of this study was to provide a basis for water environmental management decision-making. In this study, the QUAL2K model for river and stream water quality was applied to predict the water quality and environmental capacity of the Hongqi River, which is a polluted tributary in the Taihu Lake Basin. The model parameters were calibrated by trial and error until the simulated results agreed well with the observed data. The calibrated QUAL2K model was used to calculate the water environmental capacity of the Hongqi River, and the water environmental capacities of COD_Cr_ NH_3_-N, TN, and TP were 17.51 t, 1.52 t, 2.74 t and 0.37 t, respectively. The results showed that the NH_3_-N, TN, and TP pollution loads of the studied river need to be reduced by 50.96%, 44.11%, and 22.92%, respectively to satisfy the water quality objectives. Thus, additional water pollution control measures are needed to control and reduce the pollution loads in the Hongqi River watershed. The method applied in this study should provide a basis for water environmental management decision-making.

## 1. Introduction

The Taihu Lake basin is located in the provinces of Jiangsu, Zhejiang, Anhui and Shanghai and has a total area of 36,900 km^2^. This basin is the core of the Yangtze River Delta region, which has a large population and developed economy. Rapid development of the economy in the drainage area since the 1990s has resulted in increasing emissions of pollutants, which has accelerated deterioration of the water environment and eutrophication of the lake. In summer of 2007, a cyanobacteria bloom (the major species are *Microcystis*, *Anabaena* and *Aphanizomenon* of Cyanophyta) occurred in Taihu Lake and the chlorophyll-a concentrations of the water surface exceeding 100 ug/L in many areas resulted in the drinking water source for Wuxi being polluted. This incident affected the daily lives of nearly two million residents, leading to widespread concerns about water quality. From 2007 to 2010, the water quality of Taihu Lake was inferior to Grade V according to the water quality standards of China [1]. In recent years, the water quality has remained at a level of moderate eutrophication [2,3] while the nitrogen levels have been higher than the standard, especially for ammonia nitrogen. The water pollution, together with deterioration of the water ecological environment has resulted in frequent cyanobacteria blooms occurring in Taihu Lake every year from 2007, which not only affects the drinking water source, but also directly challenges the safety of the water supply to cities in the watershed. 

The pollution load of the Taihu Lake basin is primarily from the inflow rivers, which account for more than 80% of the total [4]. Therefore, the key to protection of the water environment of Taihu Lake is interception of the pollutants that discharge into Taihu Lake from various sources. The rational use of water resources has become a very important national policy issue in recent years and great efforts have been made to develop water environmental management strategies to ensure good water quality and sufficient water supply [5,6,7,8]. In this respect, water quality modeling is increasingly recognized as an effective tool for water quality management decision-making [9]. 

In recent decades, many water quality models have been developed for various types of water bodies. For example, Thayer *et al.* [10] applied a 3-dimensional model to analyze the changes of water quality in space. Spillman *et al.* [11] used a combined 3-dimensional hydrodynamic ecological model (ELCOM-CAEDYM) to elucidate the temporal and spatial variability of food supply to commercially valuable clam populations of Barbamarco Lagoon. Ning *et al.* [12] applied QUAL2E to assess the pollution prevention program for the Kao-Ping River Basin, Taiwan. Hao *et al.* [13] weighed up and distinguished the impact induced by climate change effects and human activities on stream flow changes in Xiliaohe River Basin using soil and water assessment model. In addition, QUAL2E has been applied in studies conducted by Ghosh, Drolc and Palmieri [14,15,16]. 

Pelletier *et al.* [17] confirmed the flexibility and applicability of the QUAL2K model for simulation of river water quality. Some successful examples of QUAL2K have also been published in recent years [18,19,20,21,22], for instance, Fang *et al.* [23] applied the QUAL2K model to evaluate the spatial distribution of BOD in the Qiantang River, Zhang *et al.* [24] selected the optimal water quality improvement program via simulation of various hypothetical scenarios using the QUAL2K model, so the QUAL2K model was chosen for the present study due to its popularity and ease of application. Trial and error [25,26] is a method of reaching a correct solution or satisfactory result by trying out various means or theories until errors are sufficiently reduced or eliminated. The trial and error method has been widely used in water quality models and has achieved good effects in recent years [27,28]. Thus, it is reasonable that trial and error be applied in the simulation processes of the QUAL2K model.

The Hongqi River was selected for this study because river water is used for domestic water and it is typical in many tributaries of the Wujin River, which is one of the major inflow rivers of Taihu Lake (China). In this study, the results simulated by the QUAL2K model are compared with measured data for the Hongqi River. The objective of this study is to apply the QUAL2K model to calibrate the parameters of the Hongqi River, and then simulate the water environmental capacity of the Hongqi River in accordance with the water environmental management requirements. The pollution load reduction rate was then calculated to meet the water quality objectives, which provide a basis for watershed environmental management and water quality improvement. Finally, a tool for pollution control and environmental management of the river is produced with the goal of assisting in decision-making for better use of water resources and forecasting the impending damages caused by socio-economic factors. 

## 2. Material and Methods

### 2.1. Study Area

Wujin River is situated to the north of Taihu Lake. The river is 29 km long, about 2–3 m deep and 25–30 m wide. The river flows into Meiliang Bay of Taihu Lake as the main river of the City of Changzhou and the Wujin District, as well as into Zhushan Bay through a tributary named Yapu River. Furthermore, Wujin River is the main waterway that connects Beijing-Hangzhou Grand Canal with Taihu Lake. Both Meiliang Bay and Zhushan Bay are important bays in Taihu Lake as sources of drinking water and tourist destinations. However, these bays are also the most seriously affected by cyanobacteria blooms in Taihu Lake. The water quality of Wujin River is generally inferior to the Grade V water quality standards of China [1]. The main pollutants in the river are nitrogenous nutrients and organic pollutants, among which total nitrogen and ammonia nitrogen greatly exceed the standard. Petroleum substances, dissolved oxygen and total phosphorus fail to meet the Grade IV water quality standards in some sections of the river. In recent years, the community has paid great attention to water pollution issues associated with the Wujin River. 

The Hongqi River was selected as the study area. It is one of the tributaries of the Wujin River located in the north of Taihu Lake. It runs 1.68 km between the Machi River in the west and the Wujin River in the east, and has an average width of 12.5 m, an average depth of 1.6 m, and an average annual flow velocity of 0.025 m/s. Primary sources of pollution include rural domestic sewage, industrial wastewater, farmland surface runoff, and solid waste pollution. The self-purification capability of the river is weak, partly because of the sewage emissions substantially exceed the purification capacity, and parts of the river are thus mildly eutrophic. 

**Figure 1:**
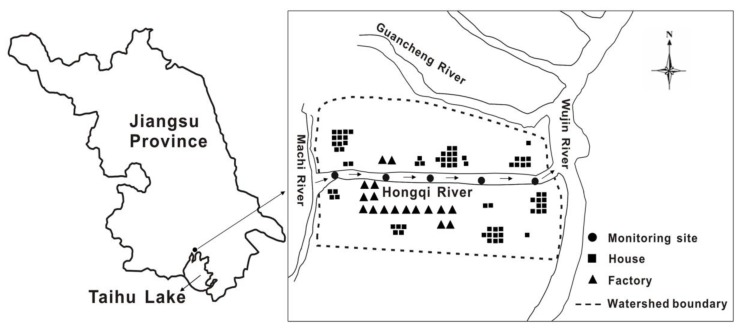
Study area and monitoring sites along the Hongqi River.

The study object included 1.5 km of the Hongqi River, with a watershed of 1.30 km^2^ (Figure 1). The arable land in the area is about 0.47 km^2^, most of which is used for paddy and vegetable cultivation, while forest and fruit trees occupy less of this area. The river is an important water source for drinking, irrigation, industry and entertainment for nearly 1,300 people. About twenty years ago, the water from the Hongqi River was drinkable after simple processing; however, rapid socioeconomic development in the area has led to increased emissions of untreated wastewater and pollutants from domestic, industrial and agricultural processes, which has resulted in decreased water quality in the river. Compared with surface water quality standards, the water quality of the Hongqi River is generally below the Grade V water quality standards [1] and the primary factors in excess are ammonia nitrogen, total nitrogen and total phosphorus.

### 2.2. Monitoring Sites and Data

In this study, five points P1–P5 along the Hongqi River were selected as monitoring sites (Figure 1). The distances from the monitoring sites to the downstream boundary are shown in Table 1.

**Table 1:** Monitoring sites along the Hongqi River.

Monitoring sites	P1	P2	P3	P4	P5
Location (km)	1.37	0.94	0.63	0.33	0.03

The monitoring was conducted under low flow velocity conditions. In addition, data were not collected in rainy days so that the results could be fit to the steady flow model QUAL2K. Monitoring was conducted from September to December 2009 in the winter season and March to June 2010 in the spring. The following water quality and hydraulic parameters were measured: water temperature, flow velocity, depth, dissolved oxygen (DO), chemical oxygen demand (COD), ammonia nitrogen (NH_3_-N), total nitrogen (TN), and total phosphorus (TP).

The water samples were collected, preserved, conveyed and monitored in accordance with the methods described in the Technical Specifications Requirements for Monitoring of Surface Water [29] and Waste Water and Water Quality Sampling-Technical Regulation of the Preservation and Handling of Samples [30]. The hydrodynamic and physical parameters, such as temperature, flow velocity and dissolved oxygen, were measured in the field [31]. Temperature and dissolved oxygen were monitored using portable sensors. Water velocity was measured using a current meter. Other parameters were measured in the laboratory. COD_Cr_ (the amount of oxygen required when use potassium dichromate oxidize the organic matter in 1 L sewage), ammonium nitrogen, nitrate nitrogen, total nitrogen and total phosphorus were determined using the Monitoring and Analytical Method on Water and Wastewater of China [32]. The monitoring work was conducted once a month and the average values of every four months are presented in Table 2 and Table 3. 

**Table 2:** Water quality monitoring data of Hongqi River for winter 2009.

Monitoring sites	Temp (°C)	DO (mg/L)	COD_Cr_ (mg/L)	NH_3_-N (mg/L)	TN (mg/L)	TP (mg/L)
P1	9.62	6.05	25.72	1.91	5.99	0.34
P2	9.40	5.76	24.34	1.86	5.52	0.31
P3	9.66	5.62	24.10	1.92	5.46	0.29
P4	9.93	5.68	23.99	1.93	5.37	0.29
P5	9.69	5.46	22.80	1.89	5.32	0.30

**Table 3:** Water quality monitoring data of Hongqi River for spring 2010.

Monitoring sites	Temp (°C)	DO (mg/L)	COD_Cr_ (mg/L)	NH_3_-N (mg/L)	TN (mg/L)	TP (mg/L)
P1	12.38	6.21	27.06	2.47	6.61	0.20
P2	12.32	5.81	25.73	2.40	6.19	0.18
P3	12.27	5.68	27.10	2.42	6.46	0.19
P4	12.37	5.73	25.78	2.43	6.29	0.16
P5	12.30	5.67	25.38	2.39	6.08	0.17

**Table 4:** Water quality standards of surface water [1].

Grade	COD_Cr _(mg/L)	BOD_5 _(mg/L)	NH_3_-N (mg/L)	TN (mg/L)	TP (mg/L)
Grade IV	30.0	6.0	1.0	1.5	0.3
Grade V	40.0	10.0	2.0	2.0	0.4

The monitoring results indicate that the water quality of Hongqi River decreases a little from upstream to downstream. Compared with the Grade IV water quality standards (Table 4), high concentrations of nitrogen, phosphorus and chemical oxygen demand are the primary problems associated with water quality in the Hongqi River watershed. In addition, because the flow velocity of the river is slow and the sewage emissions exceed the purification capacity a lot, mild eutrophication and high turbidity remains [33,34]. 

### 2.3. QUAL2K Model

QUAL2K is a one-dimensional river and stream water quality model that is an upgraded version of the QUAL2E model [35]. The QUAL2K framework, which was developed by the US Environmental Protection Agency, can simulate the migration and transformation of conventional pollutants. The model considers the stream as a one-dimensional channel with steady flow that is non-uniform and considers the influence of point source and non-point source pollution loads. The model also imitates changes with a user-opted time step inside of an hour within the daily cycle. In addition to being widely applied for the environmental management of relatively large rivers [12,18,19], the framework model also has several new features that make it applicable to shallow, upland and other rivers.

The QUAL2K framework includes the following new elements: QUAL2K uses unequally spaced reaches, and multiple loadings and withdrawals can be input to any reach. Denitrification is modeled as a first-order reaction that becomes pronounced under low oxygen concentrations. Sediment-water fluxes of dissolved oxygen and nutrients can be simulated internally rather than prescribed. That is, oxygen and nutrient fluxes are simulated as a function of settling particulate organic matter, reactions within the sediments, and concentrations of soluble forms in the overlying waters. The model explicitly simulates attached bottom algae. These algae have variable stoichiometry. Light extinction is calculated as a function of algae, detritus and inorganic solids. Both alkalinity and total inorganic carbon are simulated. QUAL2K allows the user to specify many of the kinetic parameters on a reach-specific basis [36]. 

QUAL2K can simulate the migration and transformation of a wide variety of constituents including dissolved oxygen, temperature, biochemical oxygen demand, organic nitrogen, ammonia nitrogen, nitrate nitrogen, total nitrogen, sediment oxygen demand, organic phosphorus, inorganic phosphorus, total phosphorus, phytoplankton and algae. The illustrations and uses of this model are described in detail in the QUAL2K user’s manual [36]. The model can also simulate some other factors, including pH, alkalinity and pathogenic bacteria. Overall, the bottom algae are indispensable for imitating shallow rivers. 

**Figure 2:**
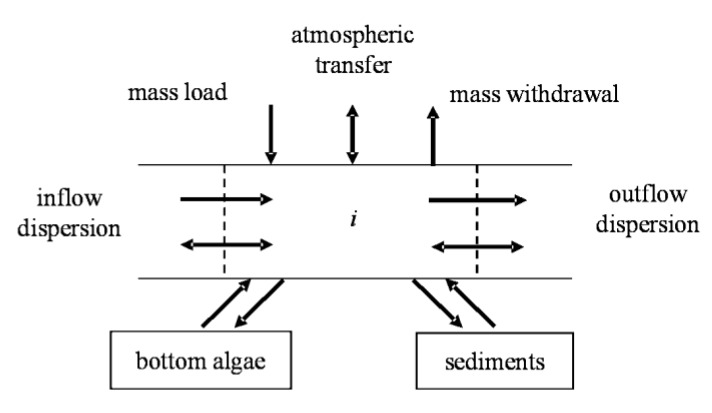
Flow diagram of the mass balance for relevant components of the river system in reach *i*.

For all but the bottom algae variables, a general mass balance for a component concentration *c_i_* (Figure 2) in the reach *i* is written as [36]:

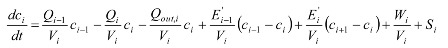
(1)


In the above formula, *c_i_*, *Q_i_*, *V_i_*, *E_i_*, and *W_i_* symbolize the component concentration of water quality, flow, volume, dispersion coefficient, and outer component load of reach *i*, respectively. *S_i_* symbolizes the sinks and sources of the component due to a large number of transformation mechanisms and reactions in reach *i*. *Q_out,i_* symbolizes flow abstraction from reach *i*. 

## 3. Application of QUAL2K to Simulate the Hongqi River

### 3.1. Input Data

**Figure 3:**
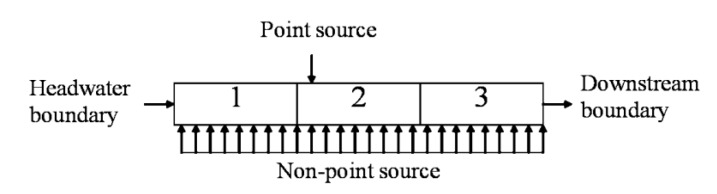
Schematic representation of the three reaches of the Hongqi River, and their sources of pollution.

According to hydrological and hydraulic conditions, locations of water quality monitoring sites, and distributions of pollution sources, the 1.5 km length of the Hongqi River was divided into three reaches, each with a length of 0.5 km. The latitude and longitude of each reach’s downstream are (31°35'35.3"N, 120°2'26.45"E), (31°35'35.1"N, 120°2'49.55"E) and (31°35'35.74"N, 120°3'6.28"E), respectively. There are 15 computational elements with a length of 100 m. Figure 3 shows the river reach with the locations of point sources of the Hongqi River. Table 5 shows the three reaches with different hydraulic characteristics. 

**Table 5:** Hydraulic characteristics of the three reaches of the Hongqi River. “Location” refers to the distance from the river’s end.

Location (km)	Flow (m^3^/s)		Depth (m)	Flow velocity (m/s)	Travel time (d)
	Winter	Spring			
1.340	0.446	0.485	1.586	0.024	0.071
0.650	0.452	0.496	1.604	0.025	0.405
0.150	0.465	0.504	1.618	0.026	0.694

Based on the method provided by National Water Environment Capacity Verification Manual [37] and combined with the data obtained from the local environmental management agency, the pollutants emissions were calculated [38]. The average annual domestic sewage emissions in the watershed are 90 L per person per day. It is estimated that the annual domestic sewage emissions in the drainage area are 41,719.5 t, of which COD_Cr_ is 7.51 t, NH_3_-N is 2.09 t and TP is 0.25 t. There is no livestock or poultry breeding in the area, so agricultural non-point source emissions are primarily from farmland. It is estimated that agricultural non-point source emissions of COD_Cr_ are 0.14 t, while those of NH_3_-N are 0.14 t, those of TN are 0.28 t and those of TP are 0.03 t per year in the drainage area. There were approximately twenty factories and enterprises in the drainage area, all of which but the Hongbo Paint Company are mechanical processing enterprises. Therefore, few pollutants are discharged into the river. It is estimated that the industrial wastewater emissions in the drainage area are 9,780 t and the COD_Cr_ is 20.5 t. The flow and concentration of pollution sources are shown in Table 6. 

**Table 6:** Flow and concentration of pollution sources.

Pollution sources	Flow (m^3^/s)	COD_Cr_ (mg/L)	NH_3_-N (mg/L)	NO_3_-N (mg/L)	TN (mg/L)	TP (mg/L)	Inorganic phosphorus (mg/L)
Non-point sources	0.00143	180	50	60	120	6	2.5
Point sources	0.00031	2,100	100	90	250	13	5.5

The input parameters involved in QUAL2K were temperature, flow velocity, COD_Cr_, dissolved oxygen, ammonium nitrogen, nitrate nitrogen, organic nitrogen, inorganic phosphorus and organic phosphorus. The level of phytoplankton in the Hongqi River is negligible. According to field survey and hydraulic characteristics of the river, the bottom algae coverage and bottom SOD coverage were determined to be 70% and 100%, respectively. 

### 3.2. Parameters

The extent of parameters (Table 7) that QUAL2K demanded were determined from a large number of studies including documentation for the stream water quality model QUAL2E [21,35], the QUAL2K user manual [36,39] and the Environment Protection Agency guidance document [40]. The internal calculation method was applied to calculate the re-aeration rate [36,41].

**Table 7:** Calibrated parameters for simulating the water quality of Hongqi River.

Parameter	Value	Units	Symbol	Range
Carbon	40	gC	gC	30–50
Nitrogen	7.2	gN	gN	3–9
Phosphorus	1	gP	gP	0.4–2
Dry weight	100	gD	gD	100
Chlorophyll	1	gA	gA	0.4–2
ISS settling velocity	1	m/day	*v_i_*	0–2
O_2_ reaeration model	Internal			
BOD hydrolysis rate	0.1	day^−1^	*k_hc_*	0.04–4.2
COD oxidation rate	0.2	day^−1^	*k_dc_*	0.02–4.2
Organic N hydrolysis	0.2	day^−1^	*k_hn_*	0.02–0.4
Organic N settling velocity	0.05	m/day	*v_on_*	0.001–0.1
Ammonium nitrification	0.5	day^−1^	*k_na_*	0–10
Nitrate denitrification	0.8	day^−1^	*k_dn_*	0–2
Sed. denitrification transfer coeff.	1.0	m/day	*v_di_*	0–1
Organic P hydrolysis	0.2	day^−1^	*k_hp_*	0–5
Organic P settling velocity	1.0	m/day	*v_op_*	0–2
Inorganic P settling velocity	0.5	m/day	*v_ip_*	0–2
Sed. P oxygen attenuation half sat constant	1.8	mgO_2_/L	*k_spi_*	0–2
Bottom algae				
Growth model	zero–order			
Max Growth rate	60	mgA/m^2^/day	*C_gb_*	0–500
First–order model carrying capacity	1,000	mgA/m2	*a_b,max_*	1,000
Respiration rate	0.25	day^−1^	*k_rb_*	0.05–0.5
Excretion rate	0.5	day^−1^	*k_eb_*	0–0.5
Death rate	0.25	day^−1^	*k_db_*	0–0.5

The exponential model was selected for oxygen inhibition of BOD hydrolysis, COD oxidation, de-nitrification, nitrification, algae and phytoplankton respiration. The influence of wind was assumed to be ignored. There are six degradation parameters including COD oxidation rate (*k_dc_*), ammonium nitrification rate (*k_na_*), nitrate denitrification rate (*k_dn_*), organic N hydrolysis rate (*k_hn_*), organic *P* hydrolysis rate (*k_hp_*), and inorganic P settling velocity rate (*k_ip_*) were obtained by trial and error. The remaining parameters were set by the default values (Table 7) in the QUAL2K model [42,43].

### 3.3. Implementation of the Model

The QUAL2K model has greater flexibility, which can follow the specific circumstances of users to set the parameter values and transform the simulation equation, satisfying user requirements for water quality simulation. In this study, the parameters of *k_hc_, k_dn_, k_dt_* (Detritus Dissolution rate) were set to 0 and *F_oxc_* (CBOD decay rate of rapid reaction at low dissolved oxygen conditions) was set to 1, so CBOD_f_ (CBOD of rapid reaction) represents the concentration of COD. The *k_dc_* was then set as the COD comprehensive degradation coefficient; thus, QUAL2K can be used to simulate the changes of COD [36].

The monitoring data for the winter of 2009 were applied for calibration. The calculation time step was set to 5.6 min to ensure the model was maintained in the steady-state. The model was run with another completely different data set, which was set without altering the calibrated parameters, so that the ability of the calibrated model to forecast the component concentration under different circumstances could be examined. Thus, the model was utilized in the future simulation.

## 4. Results and Discussion

**Figure 4:**
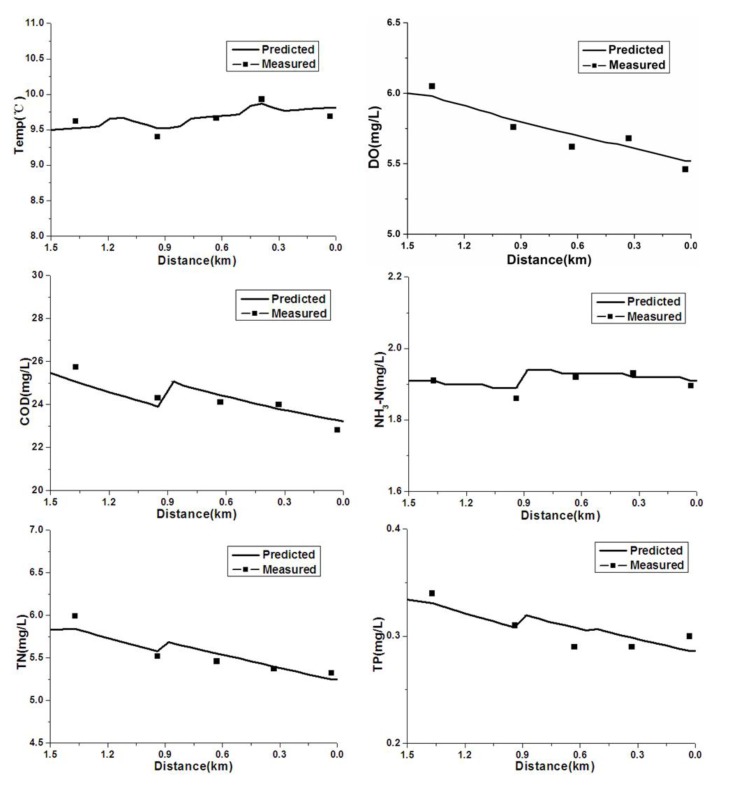
Water quality calibration results for the Hongqi River.

**Figure 5:**
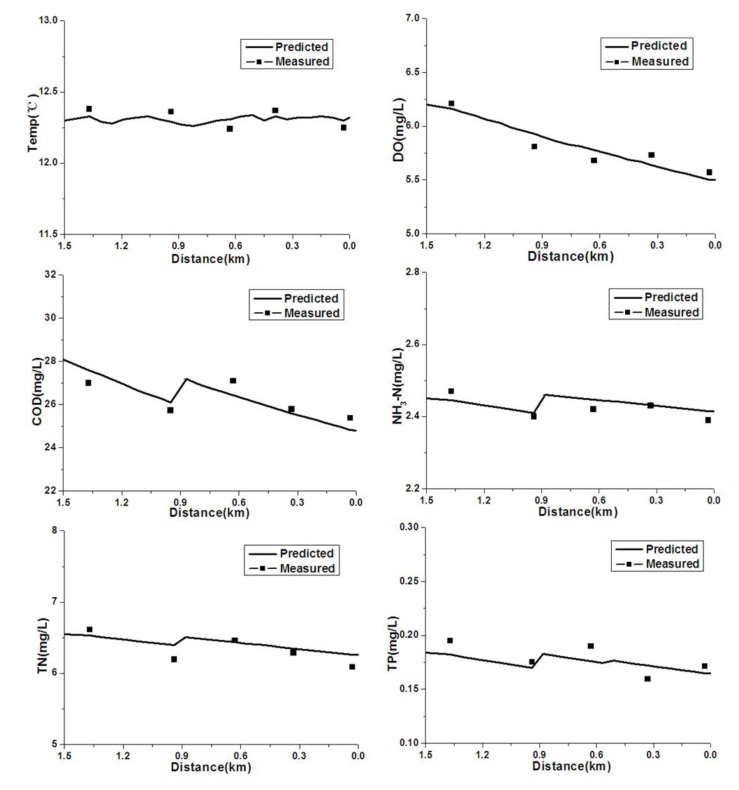
Water quality confirmation results for the Hongqi River.

The monitoring data for the water quality parameters are displayed in Table 2 (Water quality monitoring data for winter 2009), Table 3 (Water quality monitoring data for spring 2010) and Table 6 (Flow and concentration of pollution sources). Figure 4 and Figure 5 display the calibration and confirmation results, respectively. 

### 4.1. Calibration and Verification

As shown in Figure 4, the water quality improved from the headwater to the downstream areas. The concentrations of dissolved oxygen in the Hongqi River meet the Grade III DO standards [1]. Throughout the river, the concentration of DO was greater than 5 mg/L, which indicates good water quality because it is better than the water quality objectives. Since the decomposition of pollutants consumes large amounts of dissolved oxygen, the DO is reduced. In addition, the decrease in DO concentrations was due in part to the discharge of organic pollutants by the Hongbo Paint Company, which adds high levels of organics, nitrogen substances and low DO wastewater to the Hongqi River.

The concentrations of COD, NH_3_-N, TN and TP increased slightly at 0.9 km due to discharge of the local wastewater and point source pollution, which corresponds to the location of the Hongbo Paint Company. The temperature in the winter of 2009 increased from the headwater to the downstream portions of the river.

The calibration results of the QUAL2K model were in accordance with the monitoring values, with a few exceptions. For example, the simulated curves of dissolved oxygen (DO) and TP deviated slightly from the observed values. The calibrated parameters are shown in Table 7. The model was confirmed with water quality monitoring data from spring of 2010 using parameters that were calibrated based on monitoring data from the winter of 2009. The confirmation results (Figure 5) showed that the calibrated parameters are very dependable. 

From Figure 5, the observed values of DO were higher or lower than the simulated values, and the observed values of TP at the location of 0.3, 0.6, and 1.4 were deviated slightly from the simulated results. The standard deviation (SD) of temperature, DO, COD, NH_3_-N, TN and TP were 0.057, 0.129, 0.461, 0.020, 0.126, and 0.015, respectively. The relative standard deviation (RSD) of temperature, DO, COD, NH_3_-N, TN and TP were 0.005, 0.021, 0.018, 0.008, 0.019, and 0.078, respectively. The relative standard deviation of TP was the largest in these factors. Some errors in this modeling are unavoidable because the fieldwork involved gathering a water sample at each monitoring point. Nevertheless, the simulation results were acceptable to realize water environmental management targets under the conditions of limited data.

### 4.2. Calculation of the Water Environmental Capacity

#### 4.2.1. Calculation of Pollution Load

We conducted a pollution load investigation and analysis of the Hongqi River drainage area. Specifically, we studied the states of water environmental quality, the emissions of industrial wastewater, rural domestic sewage, and agricultural non-point source emissions. Based on the results, the average annual emissions of COD_Cr_, NH_3_-N, TN, and TP were calculated to be 108.47 t, 7.26 t, 16.31 t, and 1.07 t, respectively. The emissions from the headwater and the studied river reach are shown in Table 9. The proportions of COD_Cr_, NH_3_-N, TN, and TP pollution loads discharged from the headwater and local river reach were 3.99, 1.00, 2.10, and 1.23, respectively. 

#### 4.2.2. Simulation Method

**Figure 6:**
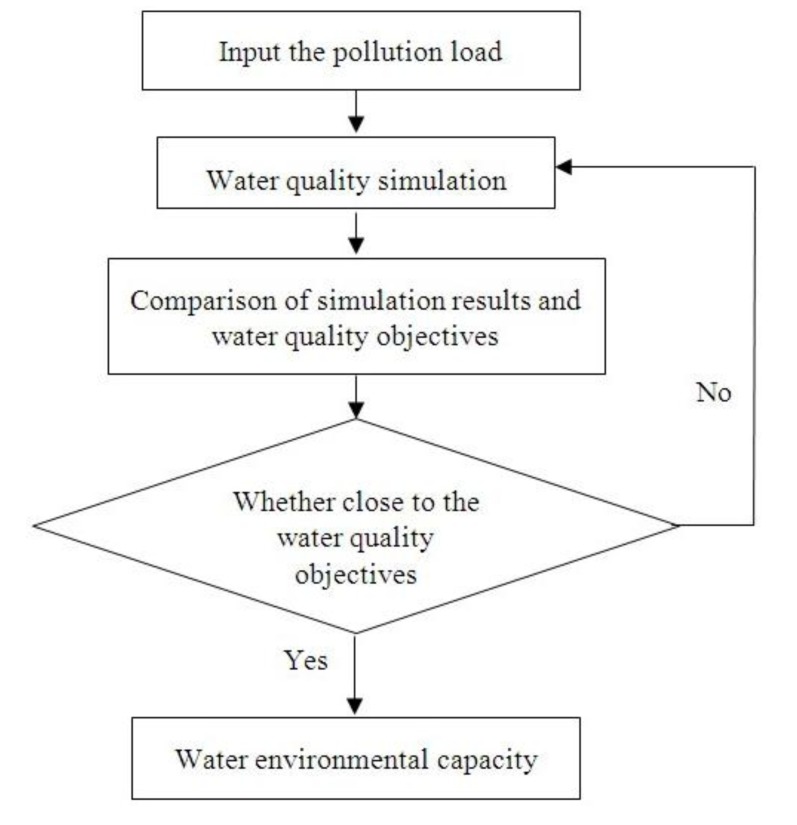
Schematic diagram of the trial and error method.

The water environmental capacity of the Hongqi River was calculated by the calibrated QUAL2K model (calibrated parameters in Table 7) using the trial and error method [26,27,28]. Specifically, the input pollution loads of the chemical oxygen demand, ammonia nitrogen, total nitrogen, and total phosphorus were adjusted by trial and error until the water quality simulation results met the water quality objectives [44]. Figure 6 shows the schematic diagram of the trial and error method.

The simulation steps are as follows:

(1) The water quality objectives must be determined based on the water environmental management requirements of the Hongqi River. According to the water environmental management requirements of Jiangsu Province, the water quality objectives of the Hongqi River watershed are Grade IV water quality standards [1]. In this study, the end of the river was the water quality control section. The water quality objectives of the Hongqi River are shown in Table 8.

**Table 8:** Water quality objectives of the Hongqi River.

Factors	COD_Cr_	NH_3_-N	TN	TP
Concentration (mg/L)	30.0	1.0	1.5	0.3

(2) To simulate the pollution loads and obtain various pollutant environmental capacities of Hongqi River, we selected the water quality requirements for the water quality control section above as the simulation objectives and adjusted the input pollution loads of COD_Cr_, ammonia nitrogen, total nitrogen, and total phosphorus. 

(3) To calculate the water environmental capacity of the studied river reach, the water quality of the headwater was set to Grade IV, and the pollutants discharged into this river reach from both shores were considered. 

#### 4.2.3. Simulation Results

**Figure 7:**
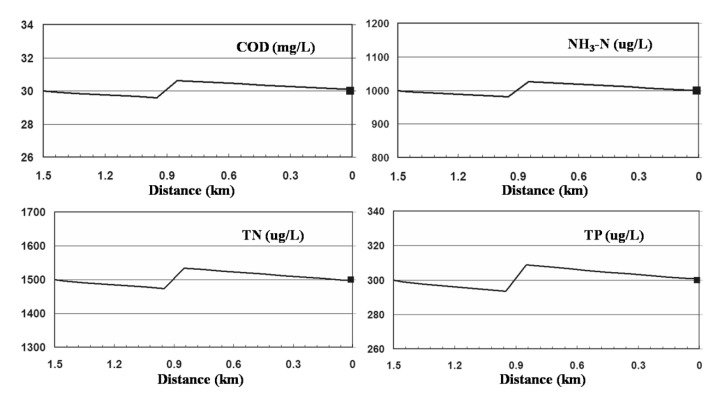
Simulation curve of water environmental capacity.

The simulation results of the pollution loads are shown in Figure 7. Table 9 shows the results of the Hongqi River water environmental capacity. The water environmental capacity was obtained by multiplying the simulation results by the average annual total flow. The water environmental capacities of COD_Cr_, NH_3_-N, TN, and TP were 17.51 t, 1.52 t, 2.74 t and 0.37 t, respectively. 

### 4.3. Pollution Load Reduction

Based on the comparative results of the pollution load emissions with the water environmental capacity of the studied river reach, the pollution load reduction required to meet the water quality objectives for the river were obtained [45]. The results are shown in Table 9. 

**Table 9:** Pollution load reduction in Hongqi River.

Water quality factors	COD_Cr_	NH_3_-N	TN	TP
Total pollution load (t)	108.47	7.26	16.31	1.07
Pollution load of headwater (t)	80.32	3.63	11.05	0.59
Pollution load of headwater in Grade IV water quality (t)	85.20	2.56	4.26	0.85
Pollution load reduction in headwater (t)	−4.88	1.07	6.79	−0.26
Pollution load reduction rate of headwater (%)	−6.08	29.48	61.45	−44.07
Pollution load of studied river reach (t)	28.15	3.63	5.26	0.48
Water environmental capacity of studied river reach (t)	17.51	1.52	2.74	0.37
Pollution load reduction in the studied river reach (t)	10.64	1.85	2.32	0.11
Pollution load reduction rate of the studied river reach (%)	37.80	50.96	44.11	22.92

The pollution load reductions required to satisfy the water quality objectives were calculated by subtracting the water environmental capacity from the pollution load emissions of the studied river reach [12]. Positive values indicate that the pollution load exceeds the environmental capacity and needs to be reduced, negative values indicate that the environmental capacity remains in surplus and can accommodate a greater pollution load. Based on the data in Table 9, the COD_Cr_, NH_3_-N, TN, and TP pollution loads of the headwater need to be reduced by −4.88 t, 1.07 t, 6.79 t, and −0.26 t, respectively, to achieve Grade IV water quality objectives. According to the water quality objectives of the Hongqi River, the pollution loads of COD_Cr_, NH_3_-N and TN in the river greatly exceeded the environmental capacity. These findings show that the nitrogen pollution in the river is very serious. The pollution loads of NH_3_-N, TN and TP must be reduced by 50.96%, 44.11% and 22.92%, respectively, to satisfy the water quality objectives. Thus, water pollution control measures such as economic instruments or macrophytes purification [46,47], are required to carry out. 

## 5. Conclusions

In this study, the one-dimensional river model QUAL2K was calibrated and confirmed using data from field monitoring carried out during the winter of 2009 and spring of 2010. The simulated results correlated with the measured data quite well. The water environmental capacity of the Hongqi River was simulated by QUAL2K and found to be 17.51 t, 1.52 t, 2.74 t and 0.37 t for COD_Cr_, NH_3_-N, TN, and TP, respectively. The pollution load reductions of NH_3_-N, TN, and TP required to meet the water quality objectives were calculated to be 29.48%, 61.45%, and −44.07% for the headwater and 50.96%, 44.11% and 22.92% for the studied river reach, respectively. Therefore, economic instruments or macrophytes purification are required to control pollution loads in the Hongqi River watershed in the long run.

The primary goal of this study was achieved in that the calibration was available for simulation of the water environmental capacity of the Hongqi River, which allowed confirmation of the parameters by the second data sets. The results of this study should provide a basis for water environmental management strategies that will be taken on by the government. 

## References

[1] State Environmental Protection Administration of the P.R. China (SEPA) (2002). Environmental Quality Standards for Surface Water(GB3838-2002).

[2] Wang H.J., Wang H.Z. (2009). Mitigation of lake eutrophication: Loosen nitrogen control and focus on phosphorus abatement. Prog. Nat. Sci..

[3] Xie P. (2008). Historical Development of Cyanobacteria with Bloom Disaster in LakeTaihu.

[4] Zhang L., Sun W., Cheng W., Liu W., Wang C. (2009). Overall treatment of water environment for inflow rivers of Lake Taihu. Environ. Monit. Manage. Technol..

[5] Eatherall A., Boorman D.B., Williams R.J., Kowe R. (1998). Modeling in-stream water quality in LOIS. Sci. Total Environ..

[6] Horn A.L., Rueda F.J., Hormann G., Fohrer N. (2004). Implementing river water quality modeling issues in mesoscale watershed models for water policy demands—An overview on current concepts, deficits, and future tasks. Phys. Chem. Earth.

[7] Mahamah D.S. (1998). Simplifying assumptions in water quality modeling. Ecol. Model..

[8] Yang M., Qian X., Zhang Y., Sheng J., Shen D., Ge Y. (2011). Spatial multicriteria decision analysis of flood risks in aging-dam management in China: A framework and case study. Int. J. Environ. Res. Public Health.

[9] Petrescu V., Sumbasacu G.O., Sirbu N. (2011). Monitoring and mathematical modeling-important tools for environmental problems. Environ. Eng. Manag. J..

[10] Thayer H.P., Krutchkoff R.G. (1967). Stochastic model for BOD and DO in streams. J. Sanit. Eng. Div..

[11] Spillman C.M., Hamilton D.P., Hipsey M.R., Imberger J. (2008). A spatially resolved model of seasonal variations in phytoplankton and clam (Tapes philippinarum) biomass in Barbamarco Lagoon, Italy. Estuar. Coast. Shelf S..

[12] Ning S.K., Chang N., Yang L., Chen H.W., Hsu H.Y. (2001). Assessing pollution prevention program by QUAL2E simulation analysis for the Kao-Ping river Basin, Taiwan. J. Environ. Manage..

[13] Hao L., Zhang X., Gao J. (2011). Simulating human-induced changes of water resources in the upper Xiliaohe river basin, China. Environ. Eng. Manag. J..

[14] Ghosh N.C. (1996). Application of QUAL2E for water quality modeling of Kali River (UP). Indian J. Environ. Health.

[15] Drolc A., Koncan J.Z. (1999). Calibration of QUAL2E model for the Sava River (Slovenia). Water Sci. Technol..

[16] Palmieri V., de Carvalho R.J. (2006). QUAL2E model for the Corumbatai River. Ecol. Model..

[17] Pelletier G.J., Chapra S.C., Tao H. (2006). QUAL2Kw—A framework for modeling water quality in stream and rivers using a genetic algorithm for calibration. Environ. Modell. Softw..

[18] Anh D.T., Bonnet M.P., Vachaud G., Minh C.V., Prieur N., Duc L.V., Anh L.L. (2006). Biochemical modeling of the Nhue River (Hanoi, Vietnam): Practical identifiability analysis and parameter estimation. Ecol. Model..

[19] Cho J.H., Ha S.R. (2010). Parameter optimization of the QUAL2K model for a multiple-reach river using an influence coefficient algorithm. Sci. Total Environ..

[20] Fan C., Ko C.H., Wang W.S. (2009). An innovative modeling approach using QUAL2K and HEC-RAS integration to assess the impact of tidal effect on river water quality simulation. J. Environ. Manage..

[21] Kim D., Wang Q., Soriala G.A., Dionysioua D.D., Timberlakeb D. (2004). A model approach for evaluating effects of remedial actions on mercury speciation and transport in a lake system. Sci. Total Environ..

[22] Park S.S., Lee Y.S. (2002). Water quality modeling study of the Nakdong river, Korea. Ecol. Model..

[23] Fang X., Zhang J., Chen Y., Xu X. (2008). QUAL2K model used in the water quality assessment of Qiantang river, China. Water Environ. Res..

[24] Zhang R., Qian X., Li H., Yuan X., Ye R. (2012). Selection of optimal river water quality improvement programs using QUAL2K: A case study of Taihu Lake Basin, China. Sci. Total Environ..

[25] Černý V. (1985). Thermodynamical approach to the traveling salesman problem: An efficient simulation algorithm. J. Optimiz. Theory App..

[26] Putnam H. (1965). Trial and error predicates and the solution to a problem of Mostowski. J. Symbolic Logic..

[27] Refsgaard J.C., Knudsen J. (1996). Operational validation and intercomparison of different types of hydrological models. Water Resour. Res..

[28] Sin G., de Pauw D.J.W., Weijers S., Vanrolleghem P.A. (2008). An efficient approach to automate the manual trial and error calibration of activated sludge models. Biotechnol. Bioeng..

[29] State Environmental Protection Administration of the P.R. China (SEPA) (2002). Technical Specifications Requirements for Monitoring of Surface Water and Waste Water (HJ/T 91-2002).

[30] Ministry of Environmental Protection of the P.R. China (MEPC) (2009). Water Quality Sampling-Technical Regulation of The Preservation and Handling of Samples (HJ 493-2009).

[31] Liu W.C., Liu S.Y., Hsu M.S., Kuo A.Y. (2005). Water quality modeling to determine minimum in stream flow for fish survival in tidal rivers. J. Environ. Manage..

[32] State Environmental Protection Administration of the P.R. China (SEPA), Water and Wastewater Monitoring and Analysis Association of the P.R. China (WWMAA) (2002). Monitoring and Analytical Method on Water and Wastewater.

[33] Li Z.K., Zhang X.J., Yang Z.Y. (2009). Ecological engineering experiment for Jinshan Lake in Zhenjiang base on techniques of immobilized nitrogen cycling bacteria. Environ. Sci..

[34] Tian M., Zhang Y.C. (2006). Experimental study on permeable dam technique to control rural non-point pollution in Taihu basin. Acta Sci. Circum..

[35] Brown L.C., Barnwell T.O. (1987). The Enhanced Stream Water Quality Models QUAL2E and QUAL2E-UNCAS: Documentation and User Manual; EPA/600/3-87/007.

[36] Chapra S.C., Pelletier G.J., Tao H. (2008). QUAL2K: A Modeling Framework for Simulating River and Stream Water Quality, Version 2.11: Documentation and Users Manual.

[37] Chinese Academy for Environmental Planning (CAEP) (2003). National Water Environment Capacity Verification Manual.

[38] Azzellino A., Salvetti R., Vismara R., Bonomo L. (2006). Combined use of the EPA-QUAL2E simulationmodel and factor analysis to assess the source apportionment of point andnon point loads of nutrients to surface waters. Sci. Total Environ..

[39] Chapra S.C., Pelletier G.J., Tao H. (2006). QUAL2K: A Modeling Framework for Simulating River and Stream Water Quality, Version 2.04: Documentation and Users Manual.

[40] USEPA (1985). Rates, Constants and Kinetics Formulations in Surface Water Quality.

[41] Owens M., Edwards R.W., Gibbs J.W. (1964). Some reaeration studies in streams. Int. J. Air Water Pollut..

[42] Becker L., Yeh W.W.G. (1973). Identification of multiple reach channel parameters. Water Resour. Res..

[43] Rode M., Suhr U., Wriedt G. (2007). Multi-objective calibration of a river water quality model-informationcontent of calibration data. Ecol. Model..

[44] Elshorbagy A., Ormsbee L. (2006). Object-oriented modeling approach to surface water quality management. Environ. Modell. Softw..

[45] Edwards A.M.C., Freestone R.J., Crockett C.P. (1997). River management in the Humber catchment. Sci. Total Environ..

[46] Luo G.Y., Zheng J.F., Xu X.Y., Cao J., Shu W.Q. (2009). Comparison of the growth characteristics and nutrient up take of four kinds of plants cultivated on a floating-bed. Acta Sci. Circum..

[47] Zhao Y., Li D.S., Luan X.L., Qiang Y.Y. (2008). Study on purified efficiency of main pollutants from domestic wastewater by three macrophytes. J. Soil Water Conserv..

